# Effect of portable air filtration systems among female residents of old age home in northern India with hazardous air quality

**DOI:** 10.14814/phy2.70475

**Published:** 2025-08-04

**Authors:** Aman Ahuja, Ashwani Kumar, Rahul Rulia, Rashmi Bhardwaj, Geetika Arya, Vineela Surapaneni, Vishal Raj, Jyothi Geetha Mohankumar, Samruddhi Chougale, Dhruva Chaudhry, Pawan Kumar Singh

**Affiliations:** ^1^ Department of Pulmonary and Critical Care Medicine Pt. B.D. Sharma Post Graduate Institute of Medical Sciences Rohtak India; ^2^ Department of Cardiology Pt. B.D. Sharma Post Graduate Institute of Medical Sciences Rohtak India; ^3^ Centre for Medical Biotechnology Maharishi Dayanand University Rohtak India; ^4^ Department of Anatomy Pt. B.D. Sharma Post Graduate Institute of Medical Sciences Rohtak India

**Keywords:** air pollution, air purifiers, blood pressure, female health, PM2.5

## Abstract

Air pollution is among leading contributors toward cardiovascular diseases. This study evaluated the impact of air filtration systems (AFS) on cardiovascular and oxidative health during severe (hazardous category) pollution season. It was a single‐arm crossover single‐center study conducted at an all‐female old age home. Portable AFS with HEPA (high‐efficiency particulate air) filters were used for 2 weeks during the intervention phase followed by sham AFS for another 2 weeks (single‐blinded). Primary outcome was change in systolic blood pressure (at baseline, after AFS and after sham AFS). Other outcomes include, change in pulse wave velocity, CRP (C‐reactive protein) levels, and 8‐Oxo‐2′‐deoxyguanosine (8‐oxo‐DG). Final analysis included data from 29 subjects with mean age 65.83 ± 6.4 years. At baseline, PM2.5 levels were in hazardous category (PM2.5: 440.38 ± 44.3 μg/m^3^). With AFS, indoor PM2.5 levels came down (131.0 ± 19.2 μg/m^3^). After sham AFS, the levels rose back to the baseline (PM2.5: 414.2 ± 32.2 μg/m^3^). Primary outcome measurement revealed a drop in both systolic (*p* < 0.001) and diastolic blood pressure (*p* = 0.14) after installation of AFS. Both CRP and 8‐oxo‐DG followed a similar trend (*p* < 0.001). Average pulse wave velocity (from 10 subjects) also decreased after AFS but rose back to baseline value after sham AFS (both *p* < 0.001). Findings of this study showed that AFS though failed to normalize the quality of air but had a positive impact on cardiovascular and inflammatory parameters.

## 1 INTRODUCTION

Air pollution is a growing global health crisis. Rampant urbanization, industrial development, and population growth have synergistically deteriorated the environment, especially air quality. Despite political commitments and various schemes, efforts to curb this menace have largely been ineffective (Peng et al., 2020). India, particularly the northern region, is heavily impacted by air pollution. Man‐made factors like paddy field fires, vehicular emissions, and coal‐powered plants contribute significantly. Ecological factors such as lower winter temperatures, humidity, and low wind speeds further worsen air quality, especially from November to January (Singh et al., 2023).

Air pollution impacts multiple systems, notably cardiovascular and respiratory. It causes both acute and chronic effects, including vascular and genomic injury. High pollution areas show increased levels of DNA damage markers. Land‐use regression models and satellite images link air pollution to higher risks of lung and other cancers (Liu et al., 2023). Measuring genomic changes and their link to cancer risk is challenging in human populations, but air pollution's acute impact on vascular reactivity is immediate and easily measured. Air pollution consists of gaseous and particulate matter, with the latter extensively studied for its inflammatory and oxidative tissue damage effects (Arias‐Pérez et al., 2020). Also, its measurement has been standardized and commonly reported while assessing air quality. World Health Organization (WHO) has set up permissible upper limits for several air quality parameters (PM2.5: 5 μg/m^3^ for annual average concentration and 15 μg/m^3^ for 24‐h average concentration).

Studying the health effects of air pollution in a prospective manner presents considerable challenges, including the long latency between exposure and disease onset, seasonal fluctuations in air quality, and population mobility. Despite this, evidence strongly supports the adverse effects of air pollution, particularly on cardiovascular health and carcinogenesis. High PM2.5 levels (particles ≤2.5 μm) are associated with increased risks of cancer and cardiovascular mortality (Manisalidis et al., 2020). Despite WHO recommendations, the limits are often crossed (exponentially), especially in developing countries like India; for example, the annual average PM2.5 concentration for the year 2023 in New Delhi was 101 μg/m^3^ (Roychowdhury et al., 2023).

With the high media coverage and real‐time AQI monitoring, air filtration systems (AFS), also known as air purifiers, are being widely used to mitigate adverse health effects. AFS generally operate on two principles: HEPA filters (filtration efficiency >99% for particulate matter up to 0.3 μm) and electrostatic filters (filtration efficiency 60%–90%). HEPA filters trap, while electrostatic filters use static energy to capture oppositely charged particles. The effectiveness of AFS is measured by their ability to entrap particulate matter; however, their efficiency in capturing and reducing volatile organic compounds (VOCs) is limited (Szczotko et al., 2022).

Previous studies have shown that AFS effectively reduce ambient PM2.5 levels (Guo et al., 2021). Even so, the impact on cardiovascular parameters, particularly in areas with extremely severe air pollution, remains poorly studied. Guo et al.'s (2021) study recruited 24 subjects for two 48‐h sessions, 12 days apart, exposing them to either true or sham AFS with doors and windows closed. Inflammation, respiratory, cardiovascular, and coagulation parameters were measured before and after the intervention. The results showed significant reductions in inflammation parameters and PM levels, but the impact on cardiovascular parameters was not significant. In a similar study from Beijing, Shao et al. (2017) observed contrasting results as no significant change in health‐related outcomes was seen between active and sham AFS, despite the 73% reduction in indoor PM2.5 levels. A similar study from Detroit, evaluated the use of high‐efficiency (HE) and low‐efficiency (LE) HEPA filters against sham AFS, for 72 h period. During the intervention indoor PM2.5 levels were significantly reduced by the AFS devices from 17.5 to 8.4 μg/m^3^ (LE) and 7.1 μg/m^3^ (HE). Investigator demonstrated a significant fall of approximately 3 mmHg in systolic blood pressure (primary end point) (Morishita et al., 2018).

In India, air pollution is severe. To our knowledge, no studies have assessed the impact of AFS on such high (hazardous category) PM2.5 levels or the subsequent health improvements. Our research aimed to evaluate the effect of air purifiers on cardiovascular variables and DNA damage during the Diwali season, as Diwali sees a rapid deterioration in air quality due to various (man‐made and environmental) factors (Ghosh et al., 2024).

## 2 MATERIALS AND METHODS

### 2.1 Study setting and design

The study was conducted in northern Indian city (Rohtak, Haryana). The subject population was from an old age home for females. The study was cleared by the institutional review board and biomedical research and ethics committee of the institute (Pandit Bhagwat Dayal Sharma Post Graduate Institute of Medical Sciences, Rohtak) vide letter number UHSR/RPSAC/2023/148‐149 dated October 13, 2023. It was a prospective single‐arm, interventional, crossover study conducted from November 2023 to January 2024. Data analysis, serological analysis, and pulse wave velocity were tested at the nearby public sector university teaching hospital.

### 2.2 Study participants

The old age home used in the study shelters homeless females. There is no age requirement for residency, but we selected subjects over 60 years old for the study. Residents typically share a room with three to four others in a 12 × 30 feet space with one door and two windows on opposite walls. They sleep for an average of 8–10 hours at night and spend the rest of their time in common areas for kitchen, laundry, cleaning, watching TV, reading, and other hobbies. We excluded only active smokers and those having an active infection, and informed the manager and caretakers not to change allocated rooms during the study. As the study investigated the cardiovascular impact of short‐term use of AFS, we did not exclude cases who were suffering from any comorbidities like obesity, hypertension, and diabetes mellitus.

### 2.3 Ethical consideration

The study was planned to investigate the impact of air purifiers on a group of elderly subjects exposed to the same quality of air (with or without air filtration systems) over a minimum 4‐week period. Such a setting could have been possible only in group living settings like old age homes and hospices. The population of hospices was influenced by several variables related to underlying disease conditions; therefore, we considered collaborating with an old age home facility. The study was designed after detailed consultations with the authorities of the old age homes, which also included some of the residents. Upon approval, the final protocol was submitted to the institutional review board and later to the biomedical research and ethics committee. For the informed consent requirements, each resident was provided a copy of the Hindi language consent form and participant information sheet. The proposed informed consent process was presented at the BREC meeting. For females who were not formally educated, an impartial witness was used. As all consents were taken from individual participants (with or without impartial witness), BREC did not mandate the use of audiovisual (AV) consenting. All interventions (installation of AFS, blood sampling, and vital examination) were done in the presence of the staff and principal investigators. For pulse wave velocity estimation, a subgroup of participants was taken to the hospital for which a separate consent was obtained. The caretaker accompanied the subjects to the hospital. All informed consents and assents processes were documented in the consent form narrations.

### 2.4 Study procedures

Following consent, 31 residents from 8 rooms were selected. Baseline assessments, including anthropometric parameters (height and weight), vitals (blood pressure, pulse rate, respiratory rate, and oxygen saturation), serum CRP, and 8‐oxo‐DG, were conducted. Ten individuals were transported to the hospital for pulse wave velocity measurement via 2D echocardiography on the same day and time. Air quality monitoring during transport was not deemed necessary, as participants were typically outdoors throughout the daytime. Portable AFS and air quality monitors were installed in each room. Residents were encouraged, but not mandated, to keep doors and windows closed during sleep. AFS and monitors were kept on continuously (for 2‐week period, electricity backup was available and both devices were auto‐start), with daily random inspections made to ensure compliance.

Fifteen days (+5 window period) post‐installation, vitals, serum CRP, and 8‐oxo‐DG were reassessed, and pulse wave velocity was measured again. After second assessments, the filters in the AFS were taken off, to begin the sham filtration phase of the study. Fifteen days after the onset of sham filtration, the same assessments were repeated. Data from air quality monitors, averaged over the preceding 5 days, were used for analysis. Participants and investigators making assessments (blood pressure, CRP, and 8‐oxo‐DG levels and pulse wave velocity) were blinded to the sham phase.

### 2.5 Study intervention (air filtration systems, pulse wave velocity measurement, exposure assessment, 8‐oxo‐DG)

#### 2.5.1 Air filtration systems

We used brand new Eureka Forbes Air Purifier 355® (model: AP 355), equipped with activated carbon, plasma, HEPA (new), and sieve filters, ideal for 480 sq. ft. rooms. The H13 HEPA filter clears 99.97% of PM0.1 particles. It operates noiselessly and adjusts fan speed based on real‐time PM2.5 levels. For sham filtrations, HEPA filters were removed from the AFS in phase two.

#### 2.5.2 Pulse wave velocity

The pulse wave velocity was measured using 2D echocardiography by calculating the distance between the QRS complex on the ECG waveform and the onset of the Doppler waveform at the distal aortic arch and left external iliac artery. The distance between points was measured over the body surface with a flexible tape, and velocity was calculated by dividing distance by transit time difference. The detailed methodology has been described previously (Styczynski et al., 2016).

#### 2.5.3 Exposure measurement

Ambient air quality was monitored using SMILEDRIVE® portable devices (model: Multi‐Gas Monitor HCHO/TVOC/PMs), which use fans and laser sensors to report PM2.5 levels. We recorded PM2.5 levels and averaged the previous 5 days' readings for analysis. All air monitors were brand new and calibrated from the company.

#### 2.5.4 Serum assessments

Serum samples were separated by centrifuge on the same day of collection and stored at −80°C. Quantitative CRP was measured using the ELISA method (from Q‐line Biotech Ltd. Batch number: QLCR230601). Human 8‐oxo‐2′‐deoxyguanosine levels were measured using ELISA kits (ELK8533) by ELK® Biotechnology (Batch number: ELK10848‐201230‐A1), following the manufacturer recommendations. Concentrations were determined by comparing the OD of samples to the standard curve.

### 2.6 Data analysis

No formal sample size calculation was attempted as the study population was restricted to one single facility. Data were collected in paper format and entered into an Excel sheet on the same day where the data were curated and coded. Clinical, anthropometric, and demographic data were recorded and analyzed. Continuous data were reported using means (with standard deviation) and medians (interquartile range), whereas categorical data were reported as numbers and percentages. For comparison of the change in blood pressure parameters, pulse wave velocity, and serum biomarkers at different time points, we used paired *t*‐test, whereas for establishing the correlation between these measurements and PM2.5 levels at different time points, we used correlation analysis. Statistical analysis was performed on the Statistical Package for Social Sciences (SPSS®) version 26 by IBM. CONSORT guidelines by EQUATOR Network were adhered to during the writing of this manuscript (Schulz et al., 2010).

## 3 RESULTS

Old age home for females was selected for the study for the reasons related to the distance from hospital, logistics, and accessibility. At baseline, 31 subjects were identified for data collection, but during the study period, two subjects were relocated to other places; hence, the final analysis included data from 29 subjects. Over a period of 3 months, three assessment visits were made. Baseline clinical and anthropodermic data are presented in Table 1. All subjects were females with a mean age of 65.83 ± 4.4 years.

**TABLE 1:** Baseline demographic variables of the study population (all females) at Visit 1.

Variables		Study population (*n* = 29); mean ± SD or *n* (%)*
Age (years)		65.83 ± 4.4
Weight (kg)		56.02 ± 12.1
BMI (kg/m^2^)		24.96 ± 5.0
Smokers (%)		4 (13.8)*
Comorbidities	DM (%)	10 (34.5)*
	HTN (%)	11 (37.9)*
	CAD (%)	3 (10.3)*
Baseline blood pressure (±SD)	Mean SBP (mmHg)	134.69 ± 20.7
	Mean DBP (mmHg)	84.28 ± 13.8
	Mean MAP (mmHg)	101.08 ± 15.2
Pulse rate (mean ± SD) (per minute)		85.72 ± 9.7
Baselines air pollution parameters	PM2.5 (μg/m^3^)	440.38 ± 44.3
	PM10 (μg/m^3^)	250.83 ± 23.5

Abbreviations: BMI, body mass index; CAD, coronary artery disease; DBP, diastolic blood pressure; DM, type II diabetes mellitus; HTN, hypertension; MAP, mean arterial pressure; PM10, particulate matter of diameter 10 μm or less; PM2.5, particulate matter of diameter 2.5 μm or less; SBP, systolic blood pressure; SD, standard deviation.

* The values whihc carry start are reported in numbers with percentages‐ *n*(%).

Most subjects were overweight (>22.9 kg/m^2^) as per Asian nutrition status criteria for BMI.

At the baseline, the air pollution parameters as per US Environmental Protection Agency categories belonged to the hazardous category (PM2.5 levels 301+, absolute value: 440.38 ± 44.3 μg/m^3^); however, with the use of air filtration systems, indoor PM2.5 levels came down drastically (131.0 ± 19.2 μg/m^3^) but still did not reach the WHO recommended limits. Upon taking off the AFS, the levels rose back to the baseline in Visit 3 (PM2.5: 414.2 ± 32.2 μg/m^3^). The same trend was also observed for PM10 (PM10, Visit 1—259.83 ± 23.5 μg/m^3^; Visit 2—51.83 ± 10.6; Visit 3—241.48 ± 34.1 μg/m^3^).

Primary outcome measurement revealed a drop in both systolic and diastolic blood pressure after installation of air purifiers. Also, after removing the filters from air purifiers, Visit 3 showed a rise in systolic blood pressure (albeit not statistically significant), whereas diastolic blood pressure remained similar to Visit 2 (Table 2). A like trend was observed for MAP and pulse rate.

**TABLE 2:** Comparison of outcomes between the three visits.

Variables	Visit 1 (baseline); mean ± SD	Visit 2 (after use of AFS); mean ± SD	Visit 3 (after removal of AFS); mean ± SD	*p* Value (Visit 1 vs. Visit 2)	*p* Value (Visit 2 vs. Visit 3)
Systolic blood pressure	134.69 ± 20.7	129.45 ± 18.4	131.31 ± 18.7	<0.001	0.637
Diastolic blood pressure	84.28 ± 13.8	81.79 ± 10.0	82.38 ± 11.0	0.135	0.89
Mean arterial pressure	101.08 ± 15.2	97.68 ± 11.6	98.02 ± 12.4	0.014	0.907
Pulse rate	85.72 ± 9.7	81.79 ± 8.0	85.59 ± 9.0	0.012	0.015
8‐oxo‐DG	1624.15 ± 927.2	1128.77 ± 657.7	1575.16 ± 952.2	<0.001	<0.001
CRP	7.86 ± 2.7	4.17 ± 2.4	7.1 ± 2.9	<0.001	<0.001
Pulse wave velocity	10.73 ± 2.2	9.48 ± 1.3	12.13 ± 1.9	0.009	<0.001
PM2.5	440.38 ± 44.3	131 ± 19.2	414.21 ± 32.2	<0.001	<0.001
PM10	250.83 ± 23.5	51.83 ± 10.6	241.48 ± 34.1	<0.001	<0.001

*Note*: All values are mean ± SD.

Abbreviations: 8‐oxo‐DG, 8‐oxo‐2'‐deoxyguanosine; AFS, air filtration system; CRP, C‐reactive protein; PM10, particulate matter of diameter 10 μm or less; PM2.5, particulate matter of diameter 2.5 μm or less; SD, standard deviation.

Serological assessment of CRP was done in all patients; however, data for 8‐oxo‐DG were available only for 27 subjects at Visits 1 and 2 and only for 24 subjects at Visit 3. Both CRP and 8‐oxo‐DG followed a similar but more prominent trend (statistically significant fall at Visit 2 from Visit 1). Average pulse wave velocity (from 10 random subjects) also decreased at Visit 2 but rose back to above baseline value at Visit 3. Both changes (from Visits 1 to 2 and 2 to 3) were statistically significant.

While comparing the absolute changes between Visit 1 and Visit 3 from Visit 2 and Visit 3, using a paired sample *t*‐test, it was found that only the drop in systolic blood pressure at Visit 2 was statistically significant, whereas for CRP, PWV, and 8‐oxo‐DG, both drop and rise were statistically significant. However, the change in values of blood pressure (any) was not significantly different between Visits 2 and 3.

Pearson correlation was also performed between the change in values, air pollution, and subject‐related parameters. It was found that there was a significant but moderate correlation between the fall in systolic blood pressure from Visits 1 to 2 and the fall in PM2.5 levels from Visits 1 to 2 (*r* = 0.36; *p* = 0.038), the difference in CRP levels from Visits 2 to 3 and the rise in PM2.5 levels from Visits 2 to 3 (*r* = 0.35; *p* = 0.031), and the change in PWV from Visits 2 to 3 and the change in PM2.5 levels in these visits (*r* = −0.564; *p* = 0.045). A similar trend was also observed for changes in PM10 levels—change in CRP levels from Visits 1 to 2 and change in PM10 levels between Visits 1 and 2 (*r* = 0.36; *p* = 0.026), also the change in CRP from Visits 3 to 3 and change in PM10 levels in these visits (*r* = −0.42; *p* = 0.011), and between the change in 8‐oxo‐DG from Visits 2 to 3 and the change in PM10 levels among these visits (*r* = 0.42; *p* = 0.026).

Exploratory analysis also revealed that there was a significant and strong correlation between change in pulse rate and PWV between Visits 3 and 2 (*r* = 0.73; *p* = 0.016). However, there was no significant correlation between change in blood pressures and change in PWV at any visits or between change in CRP levels and 8‐oxo‐DG.

## 4 DISCUSSION

In the current study, 29 otherwise healthy elderly females were recruited to assess the impact of AFS on preventing air pollution‐related adverse cardiovascular impact. It was found that even with limited exposure to AFS, there was a statistically significant improvement in hemodynamic parameters, markers of inflammation, and DNA damage end products. All improvements were followed by a reciprocal rise in these variables after removing the AFS.

In the previous similar BIAPSY trial by Shao et al., 35 residents were randomized into two groups with active and sham air purifiers for 2 weeks, followed by a crossover. Investigators measured blood pressure, heart rates, lung functions, and inflammation parameters at two points 4 weeks apart. Unlike our findings, they observed no positive impact on clinical pulmonary or cardiovascular outcomes despite significant reductions in PM2.5, carbon content, and gaseous pollutants by AFS. This contrast may be due to differences in baseline air pollution severity (PM 2.5 levels of 90 in their study vs. 440 in current study), our study's focus on elderly women, and our dynamic parameters like PWV and BP. Supporting the results of our study, reanalysis of the BIAPSY trial, using an 8‐h moving average of blood pressure and heart rate variability, found associations with PM2.5 levels, indicating some cardiovascular benefit with AFS use (Liu et al., 2018; Shao et al., 2017).

In another study, Chen et al. studied the impact of AFS on 35 healthy college‐going adults. Results demonstrated significant improvement in PM2.5 indoor levels (baseline 96.2 μg/m^3^), markers of inflammation, and blood pressure. Though the impact was substantial, the design of the study was unpragmatic. The intervention was only for 48 h, and participants were supposed to remain indoor during the entire intervention period; therefore, it remained unknown whether part‐day exposure to AFS may continue to remain beneficial (Chen et al., 2015). Another Chinese study evaluating a 36‐h AFS intervention in similarly aged participants was also shown to have a positive impact on indoor PM2.5 levels, systolic blood pressure, and SpO_2_ (Xia et al., 2023). The same group also investigated the genomic impact of PM2.5 levels by measuring DNA methylation in long interspersed nucleotide elements and 10 specific genes between groups exposed to true and sham AFS. They observed that DNA methylation was significantly, linearly, and reversibly decreased with an increase in PM2.5 levels. The study intervention was similar as described previously (over 48 h) (Chen et al., 2016). These findings were also confirmed by another study from China where PM2.5 exposure was found to elevate cytokine levels and cause changes in DNA methylation patterns at several CpG sites (Sun et al., 2020). Long‐term effectiveness of AFS was studied in 47 elderly subjects by Xia et al., where cardiovascular parameters were measured over 1 year, with half of the subjects serving as controls. Partly like our study, a fall in blood pressure (both diastolic and systolic) was observed with the use of AFS. Contrasting to our study, baseline PM2.5 levels were significantly lower (31 μg/m^3^ in the control). Pulse wave velocity was not measured (Xia et al., 2024). Karottki et al.'s Denmark study found no significant changes in microvascular parameters, lung function, or systemic inflammation markers with AFS use among elderly subjects (baseline PM2.5 levels: 8 μg/m^3^). These conflicting results compared with our study and those from China may be due to the vast differences in PM2.5 levels across settings (Karottki et al., 2013). Morishita et al.'s interventional study in Detroit included 40 participants in three blinded scenarios (sham, low‐efficiency HEPA, high‐efficiency HEPA) over 3 days. Unlike Chen et al.'s study, there were no restrictions on outdoor activities or windows, but participants wore personal air monitors. The primary outcome was SBP, with other hemodynamic parameters as secondary outcomes. Mean indoor PM2.5 exposure was 15.5 μg/m^3^. Both HEPA AFS groups (low and high efficiency) demonstrated a drop in SBP compared with sham, more pronounced in obese subjects. All secondary outcomes improved quantitatively with AFS but weren't statistically significant. Results aligned with ours despite large PM2.5 level differences (Morishita et al., 2018). In a study from Taipei (Taiwan), long‐term impact of AFS was assessed over 2 years among 200 subjects. The investigators demonstrated that the use of AFS was associated with a statistically significant fall in SBP and DBP. Similar to our study, the paired *t*‐test also confirmed a fall in hs‐CRP and 8‐oxo‐DG levels upon use of air purifiers. Indoor PM2.5 levels in the control arm were 21.4 μg/m^3^ and in the intervention (air purifiers) arm were 12.8 μg/m^3^ (Chuang et al., 2017). However, the utility and impact of using AFS round the year remain unknown as air pollution levels vary significantly over the seasons.

In a UK office‐based study, 40 participants were tested for changes in heart rate variability and cognitive functions after 5 h of AFS exposure. Baseline PM2.5 levels were 18 μg/m^3^ (3.7 μg/m^3^ in the intervention group). Results showed significant reductions in heart rate variability with higher PM2.5 (Zhou et al., 2024). Similarly, a 2‐day vehicle study on 48 adults found that high PM2.5 levels were linked to reduced multitasking capacity, increased reaction times, and lower heart rate variability (Mallach et al., 2023).

Previous studies have also shown a consistent negative impact of high PM2.5 levels on heart rate variability among patients with COPD and coronary artery diseases, which was mitigated with short‐term use of AFS (Eom et al., 2022; Raju et al., 2023). While studies have shown a linear and detrimental acute impact of PM2.5 exposure (and positive impact of use of AFS) on cardiovascular parameters, no association has been demonstrated for pulmonary parameters with PM2.5 levels (Fong et al., 2023; Yoda et al., 2020).

The hypothetical mechanism behind our findings can be attributed to the small size of PM2.5, allowing their penetration into the alveoli and bloodstream, triggering immune activation and the release of pro‐inflammatory cytokines, such as IL‐6 and TNF‐α. This systemic inflammation possibly contributes to elevated blood pressure by promoting vasoconstriction, arterial stiffness, and increased sympathetic nervous system activity. Furthermore, PM2.5 exposure induces oxidative stress by generating reactive oxygen species (ROS), leading to the formation of DNA adducts and oxidative lesions (Lee et al., 2014; Wei et al., 2009).

Our study presents several key findings. Firstly, the health effects of severe air pollution exposure are under‐researched, as most studies focus on lower (relatively) pollution levels. We found mean PM2.5 levels in the hazardous category, common in northern India during winters, nearly 30 times the WHO's recommended 24‐h average. Even with high‐efficacy AFS, levels remained about 10 times above safe limits. Secondly, while AFS is marketed as the primary personal protection against air pollution, its optimal usage regarding time, place, and duration requires further study. Most participants used AFS only during sleep, reflecting real‐world usage. Despite strengths, our study had limitations, such as small sample size, a female‐only population, not measuring other air pollution markers, and being a single‐center experience.

Overall, this is the first study to demonstrate positive cardiovascular and metabolic impacts from 9 to 10 h of AFS usage amid hazardous air pollution. In conclusion, limited AFS exposure during the heavy pollution season showed meaningful cardiovascular benefits for elderly women in northern India. However, larger, multicentric, and long‐term studies are needed to fully understand AFS's health benefits, especially in high pollution areas.

## AUTHOR CONTRIBUTIONS

Conceptualization: AA, GA, and PKS. Methodology: PKS, AK, AA, and DC. Formal analysis: PKS, GA, and RB. Investigation: RB, RR, AK, VR, SC, and VS. Writing: JGM, PKS, and RR. Writing review: DC, JGM, and GA. Funding acquisition: PKS and DC. Resources: DC, PKS, and RR. Supervision: DC.

## FUNDING INFORMATION

This research received Intramural Funding from the institution vide letter number: RC/UHSR/2023/178‐92.

## CONFLICT OF INTEREST STATEMENT

None to declare.

## ETHICS STATEMENT

This study was conducted in accordance with the principles outlined in the Declaration of Helsinki. The study protocol was reviewed and approved by the Biomedical Research and Ethics Committee of the Institute with approval number UHSR/RPSAC/2023/148–149 dated 13‐10‐2023. Written informed consent was obtained from all participants. All data were anonymized to protect participant confidentiality.

## Data Availability

The anonymized individual patient data can be shared upon request to the corresponding author and permission of the institutional ethics committee (Pandit Bhagwat Dayal Sharma Post Graduate Institute of Medical Sciences, Rohtak).

## References

[phy270475-bib-0001] Arias‐Pérez, R. D. , Taborda, N. A. , Gómez, D. M. , Narvaez, J. F. , Porras, J. , & Hernandez, J. C. (2020). Inflammatory effects of particulate matter air pollution. Environmental Science and Pollution Research International, 27, 42390–42404. 32870429 10.1007/s11356-020-10574-w

[phy270475-bib-0002] Chen, R. , Meng, X. , Zhao, A. , Wang, C. , Yang, C. , Li, H. , Cai, J. , Zhao, Z. , & Kan, H. (2016). DNA hypomethylation and its mediation in the effects of fine particulate air pollution on cardiovascular biomarkers: A randomized crossover trial. Environment International, 94, 614–619. 27397927 10.1016/j.envint.2016.06.026

[phy270475-bib-0003] Chen, R. , Zhao, A. , Chen, H. , Zhao, Z. , Cai, J. , Wang, C. , Yang, C. , Li, H. , Xu, X. , Ha, S. , Li, T. , & Kan, H. (2015). Cardiopulmonary benefits of reducing indoor particles of outdoor origin: a randomized, double‐blind crossover trial of air purifiers. Journal of the American College of Cardiology, 65, 2279–2287. 26022815 10.1016/j.jacc.2015.03.553 PMC5360574

[phy270475-bib-0004] Chuang, H. C. , Ho, K. F. , Lin, L. Y. , Chang, T. Y. , Hong, G. B. , Ma, C. M. , Liu, I. J. , & Chuang, K. J. (2017). Long‐term indoor air conditioner filtration and cardiovascular health: A randomized crossover intervention study. Environment International, 106, 91–96. 28624750 10.1016/j.envint.2017.06.008

[phy270475-bib-0005] Eom, S. Y. , Kim, A. , Lee, J. H. , Kim, S. M. , Lee, S. Y. , Hwang, K. K. , Lim, H. J. , Cho, M. C. , Kim, Y. D. , Bae, J. W. , Kim, J. H. , & Lee, D. I. (2022). Positive effect of air purifier intervention on baroreflex sensitivity and biomarkers of oxidative stress in patients with coronary artery disease: A randomized crossover intervention trial. International Journal of Environmental Research and Public Health, 19, 7078. 35742327 10.3390/ijerph19127078 PMC9223013

[phy270475-bib-0006] Fong, W. C. G. , Kadalayil, L. , Lowther, S. , Grevatt, S. , Potter, S. , Tidbury, T. , Bennett, K. , Larsson, M. , Nicolas, F. , Kurukulaaratchy, R. , & Arshad, S. H. (2023). The efficacy of the Dyson air purifier on asthma control: A single‐center, investigator‐led, randomized, double‐blind, placebo‐controlled trial. Annals of Allergy, Asthma & Immunology, 130, 199–205. 10.1016/j.anai.2022.10.010 36288782

[phy270475-bib-0007] Ghosh, B. , Barman, H. C. , & Padhy, P. K. (2024). Trends of air pollution variations during pre‐Diwali, Diwali and post‐Diwali periods and health risk assessment using HAQI in India. Discover Environment, 2, 81.

[phy270475-bib-0008] Guo, M. , Du, C. , Li, B. , Yao, R. , Tang, Y. , Jiang, Y. , Liu, H. , Su, H. , Zhou, Y. , Wang, L. , Yang, X. , Zhou, M. , & Yu, W. (2021). Reducing particulates in indoor air can improve the circulation and cardiorespiratory health of old people: A randomized, double‐blind crossover trial of air filtration. Science of the Total Environment, 798, 149248. 34325134 10.1016/j.scitotenv.2021.149248

[phy270475-bib-0009] Karottki, D. G. , Spilak, M. , Frederiksen, M. , Gunnarsen, L. , Brauner, E. V. , Kolarik, B. , Andersen, Z. J. , Sigsgaard, T. , Barregard, L. , Strandberg, B. , Sallsten, G. , Møller, P. , & Loft, S. (2013). An indoor air filtration study in homes of elderly: cardiovascular and respiratory effects of exposure to particulate matter. Environmental Health, 12, 116. 24373585 10.1186/1476-069X-12-116 PMC3893545

[phy270475-bib-0010] Lee, M. S. , Eum, K. D. , Fang, S. C. , Rodrigues, E. G. , Modest, G. A. , & Christiani, D. C. (2014). Oxidative stress and systemic inflammation as modifiers of cardiac autonomic responses to particulate air pollution. International Journal of Cardiology, 176, 166–170. 25074558 10.1016/j.ijcard.2014.07.012 PMC4134957

[phy270475-bib-0011] Liu, C. S. , Wei, Y. , Danesh Yazdi, M. , Qiu, X. , Castro, E. , Zhu, Q. , Li, L. , Koutrakis, P. , Ekenga, C. C. , Shi, L. , & Schwartz, J. D. (2023). Long‐term association of air pollution and incidence of lung cancer among older Americans: A national study in the Medicare cohort. Environment International, 181, 108266. 37847981 10.1016/j.envint.2023.108266 PMC10691920

[phy270475-bib-0012] Liu, S. , Chen, J. , Zhao, Q. , Song, X. , Shao, D. , Meliefste, K. , Du, Y. , Wang, J. , Wang, M. , Wang, T. , Feng, B. , Wu, R. , Xu, H. , Bei, H. , Brunekreef, B. , & Huang, W. (2018). Cardiovascular benefits of short‐term indoor air filtration intervention in elderly living in Beijing: An extended analysis of BIAPSY study. Environmental Research, 167, 632–638. 30172196 10.1016/j.envres.2018.08.026

[phy270475-bib-0013] Mallach, G. , Shutt, R. , Thomson, E. M. , Valcin, F. , Kulka, R. , & Weichenthal, S. (2023). Randomized cross‐over study of in‐vehicle cabin air filtration, air pollution exposure, and acute changes to heart rate variability, saliva cortisol, and cognitive function. Environmental Science & Technology, 57, 3238–3247. 36787278 10.1021/acs.est.2c06556 PMC9979657

[phy270475-bib-0014] Manisalidis, I. , Stavropoulou, E. , Stavropoulos, A. , & Bezirtzoglou, E. (2020). Environmental and health impacts of air pollution: A review. Frontiers in Public Health, 8, 14. 32154200 10.3389/fpubh.2020.00014 PMC7044178

[phy270475-bib-0015] Morishita, M. , Adar, S. D. , D'Souza, J. , Ziemba, R. A. , Bard, R. L. , Spino, C. , & Brook, R. D. (2018). Effect of portable air filtration systems on personal exposure to fine particulate matter and blood pressure among residents in a low‐income senior facility: A randomized clinical trial. JAMA Internal Medicine, 178, 1350–1357. 30208394 10.1001/jamainternmed.2018.3308 PMC6233749

[phy270475-bib-0016] Peng, W. , Dai, H. , Guo, H. , Purohit, P. , Urpelainen, J. , Wagner, F. , Wu, Y. , & Zhang, H. (2020). The critical role of policy enforcement in achieving health, air quality, and climate benefits from India's clean electricity transition. Environmental Science & Technology, 54, 11720–11731. 32856906 10.1021/acs.est.0c01622

[phy270475-bib-0017] Raju, S. , Woo, H. , Koehler, K. , Fawzy, A. , Liu, C. , Putcha, N. , Balasubramanian, A. , Peng, R. D. , Lin, C. T. , Lemoine, C. , Wineke, J. , Berger, R. D. , Hansel, N. N. , & McCormack, M. C. (2023). Indoor air pollution and impaired cardiac autonomic function in chronic obstructive pulmonary disease. American Journal of Respiratory and Critical Care Medicine, 207, 721–730. 36288428 10.1164/rccm.202203-0523OC PMC10037475

[phy270475-bib-0018] Roychowdhury, A. , Somvanshi, A. , & Kaur, S. (2023). 2023 – The crossroad: Yearend analysis of PM2.5 pollution in Delhi. Centre for Science and Environment.

[phy270475-bib-0019] Schulz, K. F. , Altman, D. G. , & Moher, D. (2010). CONSORT 2010 statement: updated guidelines for reporting parallel group randomised trials. BMJ (Clinical Research Ed.), 340, c332. 10.1136/bmj.c332 PMC2844940 20332509

[phy270475-bib-0020] Shao, D. , Du, Y. , Liu, S. , Brunekreef, B. , Meliefste, K. , Zhao, Q. , Chen, J. , Song, X. , Wang, M. , Wang, J. , Xu, H. , Wu, R. , Wang, T. , Feng, B. , Lung, C. S. , Wang, X. , He, B. , & Huang, W. (2017). Cardiorespiratory responses of air filtration: A randomized crossover intervention trial in seniors living in Beijing: Beijing Indoor Air Purifier StudY, BIAPSY. Science of the Total Environment, 603, 541–549. 28645052 10.1016/j.scitotenv.2017.06.095

[phy270475-bib-0021] Singh, T. , Matsumi, Y. , Nakayama, T. , Hayashida, S. , Patra, P. K. , Yasutomi, N. , Kajino, M. , Yamaji, K. , Khatri, P. , Takigawa, M. , Araki, H. , Kurogi, Y. , Kuji, M. , Muramatsu, K. , Imasu, R. , Ananda, A. , Arbain, A. A. , Ravindra, K. , Bhardwaj, S. , … Mor, S. (2023). Very high particulate pollution over northwest India captured by a high‐density in situ sensor network. Scientific Reports, 13, 13201. 37580480 10.1038/s41598-023-39471-1 PMC10425363

[phy270475-bib-0022] Styczynski, G. , Rdzanek, A. , Pietrasik, A. , Kochman, J. , Huczek, Z. , Sobieraj, P. , Gaciong, Z. , & Szmigielski, C. (2016). Echocardiographic assessment of aortic pulse wave velocity: Validation against invasive pressure measurements. Journal of the American Society of Echocardiography, 29, 1109–1116. 27614541 10.1016/j.echo.2016.07.013

[phy270475-bib-0023] Sun, Y. , Huang, J. , Zhao, Y. , Xue, L. , Li, H. , Liu, Q. , Cao, H. , Peng, W. , Guo, C. , Xie, Y. , Liu, X. , Li, B. , Liu, K. , Wu, S. , & Zhang, L. (2020). Inflammatory cytokines and DNA methylation in healthy young adults exposure to fine particulate matter: A randomized, double‐blind crossover trial of air filtration. Journal of Hazardous Materials, 398, 122817. 32516725 10.1016/j.jhazmat.2020.122817

[phy270475-bib-0024] Szczotko, M. , Orych, I. , Mąka, Ł. , & Solecka, J. (2022). A review of selected types of indoor air purifiers in terms of microbial air contamination reduction. Atmosphere, 13, 800.

[phy270475-bib-0025] Wei, Y. , Han, I. K. , Shao, M. , Hu, M. , Zhang, O. J. , & Tang, X. (2009). PM2.5 constituents and oxidative DNA damage in humans. Environmental Science & Technology, 43(13), 4757–4762. 19673262 10.1021/es803337c

[phy270475-bib-0026] Xia, X. , Chan, K. H. , Kwok, T. , Wu, S. , Man, C. L. , & Ho, K. F. (2024). Effects of long‐term indoor air purification intervention on cardiovascular health in elderly: a parallel, double‐blinded randomized controlled trial in Hong Kong. Environmental Research, 247, 118284. 38253196 10.1016/j.envres.2024.118284 PMC11850294

[phy270475-bib-0027] Xia, X. , Niu, X. , Chan, K. , Xu, H. , Shen, Z. , Cao, J. J. , Wu, S. , Qiu, H. , & Ho, K. F. (2023). Effects of indoor air purification intervention on blood pressure, blood‐oxygen saturation, and heart rate variability: A double‐blinded cross‐over randomized controlled trial of healthy young adults. Science of the Total Environment, 874, 162516. 36868269 10.1016/j.scitotenv.2023.162516

[phy270475-bib-0028] Yoda, Y. , Tamura, K. , Adachi, S. , Otani, N. , Nakayama, S. F. , & Shima, M. (2020). Effects of the use of air purifier on indoor environment and respiratory system among healthy adults. International Journal of Environmental Research and Public Health, 17, 3687. 32456250 10.3390/ijerph17103687 PMC7277583

[phy270475-bib-0029] Zhou, J. , Huebner, G. , Liu, K. Y. , & Ucci, M. (2024). Heart rate variability, electrodermal activity and cognition in adults: Association with short‐term indoor PM2.5 exposure in a real‐world intervention study. Environmental Research, 263, 120245. 39490569 10.1016/j.envres.2024.120245

